# Psychological resilience and work engagement of Chinese nurses: a chain mediating model of career identity and quality of work life

**DOI:** 10.3389/fpsyg.2023.1275511

**Published:** 2023-11-16

**Authors:** Zhixing Meng, Lan Zhang, Haijing Zan, Jingru Wang

**Affiliations:** ^1^Department of Nursing, Jinzhou Medical University, Jinzhou, China; ^2^Department of Nursing, The First Affiliated Hospital of Jinzhou Medical University, Jinzhou, China

**Keywords:** psychological resilience, career identity, work engagement, quality of work life, nurses

## Abstract

**Aim:**

To investigate how nurses’ psychological resilience affects their work engagement and the resulting pathways, namely, the intermediary effect of career identity and quality of work life.

**Background:**

Psychological resilience is the ability to adapt to new circumstances and overcome difficulties. Work engagement is a positive, perfect emotional and cognitive state in the work process, which has a positive effect on nurses’ physical and mental health and career development. The importance of psychological resilience in nursing is growing in popularity. However, few studies have explored the relationship between psychological resilience and nurses’ work engagement.

**Design:**

This is a cross-sectional study.

**Methods:**

From March to April 2023, 356 nurses in the First Affiliated Hospital of Jinzhou Medical University in China received valid questionnaires. The study was surveyed using the Connor-Davidson, Resilience Scale, the Nursing Career Identity Scale, the Work-Related Quality of Life Scale, and the 15-item Utrecht Work Engagement Scale. Process version 3.5 plug-in SPSS 25 was used to test the mediating effect.

**Results:**

(1) Psychological resilience was significantly and positively correlated with career identity, quality of work life, and work engagement (*r* = 0.702–0.803, *p* < 0.001). (2) Career identity and quality of work life partially mediated the relationship between psychological resilience and work engagement, with effect sizes of 0.2382 and 0.0958, respectively. (3) There was a chain mediation model between psychological resilience and work engagement that had a value of 0.1219.

**Conclusion:**

Career identity and quality of work life played a chain-mediating role between psychological resilience and work engagement. Thus, in order to enhance the work engagement of clinical nurses, it is necessary for nursing managers to take measures to enhance not only psychological resilience but also their career identity and the quality of work life.

## 1. Introduction

Nowadays, nurses, as an important component of hospitals, account for 59% of global health professionals and play an important role in providing medical services (Cai et al., 2023). Nurses are the largest group of employees in healthcare organizations and the ones who spend the most time providing direct patient care. Their performance and quality of care are key components of a hospital’s core competencies and are critical to reducing medical errors and ensuring safe patient outcomes (Al-Hamdan and Bani Issa, 2022). However, at present, nurses are facing the challenges of a relatively insufficient number and a high turnover rate. In China, the average yearly turnover rate for nurses is 28%, which negatively impacts healthcare, especially after the outbreak of the coronavirus disease (COVID-19) (Wang et al., 2019; Catton, 2021). Recent data from the National Health and Medical Commission of China reported that there were about 5.2 million nurses in China. At the end of 2022, there were 3.7 registered nurses for every 1,000 people, with a medical-to-nursing ratio of 1:1.18. This is well below the World Health Organization average of nine nurses per 1,000 people. In China, there is a million-level shortage of nurses. Chronic nursing shortages and high turnover rates in hospitals will increase the workload of nurses and put a lot of physical and psychological pressure on existing staff (Stechmiller, 2002; Chan et al., 2013), making them less energetic and less motivated to work. How to motivate most nurses and promote the development of the nursing team has become a hot topic of current research.

Work engagement, originating in positive psychology, is a positive and complete state of integration of individuals in their work, which is manifested by energy, dedication, full attention, and a strong sense of recognition of their work (Schaufeli et al., 2002). Work engagement has a positive effect on nurses’ physical and mental health and career development, enhancing job satisfaction, improving job performance and productivity, and reducing absenteeism and turnover intentions (Simone et al., 2018; Giménez-Espert et al., 2020). Meanwhile, with a high level of work engagement, nurses are also energetic and committed at work, compassionate to patients, and do their best to help patients relieve pain, which improves the quality of care and organizational performance (Brubakk et al., 2019; Kim and Seo, 2021). As nurses are more important professionals in the field of health care, the improvement of the quality of hospital nursing services is closely related to the level of nurses’ work engagement. Understanding the status of nurses’ work engagement and thoroughly studying the influencing factors of nurses’ work engagement level is essential to promote the development of hospitals and individual nurses. Psychological resilience is the ability to adapt to new circumstances and overcome difficulties. It is accompanied by personal development and change (Connor and Davidson, 2003; Topiwala et al., 2019). Some studies have shown that people who are more psychologically resilient tend to be more engaged in their work (Cao and Chen, 2020; Lyu H. et al., 2020). Psychological resilience can be a positive predictor of increasing work engagement (Cao and Chen, 2019; Clark et al., 2021). Therefore, this research attempts to investigate the following: Hypothesis 1: psychological resilience will significantly predict work engagement (psychological resilience → work engagement).

Career identity is a complete psychological process involving cognition, emotion, and behavioral tendencies that reflect people’s understanding of their relevance and value in the workplace (ten Hoeve et al., 2014; Yu et al., 2022). Psychological resilience can have a positive impact on career identity (Yi et al., 2023). Career identity benefits from resilience. Resilience serves as a work resource that enables nurses to actively confront and effectively manage the stresses and obstacles at work, spark workplace excitement, and enhance career identity. According to research, career identity and work engagement are close relationships (Wu et al., 2022). People with a strong career identity tend to devote more time and energy to their jobs and find it easier to get positive feedback from employers (Sun et al., 2022). Therefore, this research attempts to investigate the following: Hypothesis 2: the relationship between psychological resiliency and job engagement will be mediated by professional identity (psychological resilience → career identity → work engagement).

The definition of the quality of work life is that of a worker’s satisfaction with his or her work, and it is influenced by an individual’s feelings and perceptions (Mosadeghrad et al., 2011; Al-Dossary, 2022). Studies in various demographics discovered a substantial positive correlation between employees’ work engagement and quality of work life (Kanten and Sadullah, 2012). A study showed that among nurses with standardized training, quality of work life was significantly and positively associated with work engagement (Sun et al., 2022). In addition, a research has confirmed that job stress and psychological resilience play an important role in explaining various aspects of quality of work life (Tseng et al., 2018). Psychological resilience can act as a protective factor against job stress when it reduces the quality of work life. However, there is a lack of empirical data in clinical nursing studies to support the association of nurses’ work engagement with psychological resilience and their quality of work life. Therefore, this research attempts to investigate the following: Hypothesis 3: quality of work life will act as a mediator between psychological resilience and work engagement (psychological resilience → quality of work life → work engagement).

Research has shown that high levels of psychological resilience typically allow a person to deal more effectively with his or her workplace, increase his or her commitment to work, and alleviate symptoms of burnout (Guo et al., 2018). Nurses with high psychological resilience can be combined with factors of labor psychology represented by the quality of life and positive attitudes represented by career identity to cope with stress, increase job satisfaction, and ultimately enhance work engagement. According to the Job Demands-Resources model (Bakker and Demerouti, 2007), career identity and quality of work life can be used as job resources to increase clinical nurses’ work engagement. In addition, it has been shown that there is a significant positive correlation between career identity and quality of work life (Sun et al., 2022). Therefore, this research attempts to investigate the following: Hypothesis 4: career identity and quality of work life will jointly act as a chain mediator role in the relationship between psychological resilience and work engagement (psychological resilience →career identity → quality of work life →work engagement).

Although the association between psychological resilience and work engagement has been examined, the mechanisms behind the association remain largely unknown. In particular, it is unclear whether career identity and quality of work life have a mediating role in psychological resilience and work engagement. In addition, most of the current studies on nurses’ psychological resilience and work engagement have focused on individual departments, and the analysis of influencing factors has been limited to three variables. Therefore, this study combines positive psychology theory to explore the influence of Chinese nurses’ psychological resilience on work engagement and analyzes the mediating role of career identity and quality of work life to guide nurses’ work engagement in clinical practice and promote the high-quality development of nursing teams.

## 2. Methods

### 2.1. Study aim

In this study, the relationship between psychological resilience, career identity, and quality of work life on nurses’ work engagement was first tested. Furthermore, the mediation effect of career identity and quality of work life on psychological resilience and work engagement was also discussed. In the end, the author analyses the possible chain mediating role of career identity and quality of work life in the relationship between psychological resilience and nurses’ work engagement.

### 2.2. Study design

This is a cross-sectional study. The STROBE checklist was applied as the reporting guideline for this study.

### 2.3. Sample and setting

From March 2023 to April 2023, we used a convenient sampling method to select clinical nurses from the First Affiliated Hospital of Jinzhou Medical University in China. To be eligible, all participants had to be certified registered nurses with at least a year of clinical nursing experience. All participants volunteered to participate in this study and signed an informed consent form. Practice nurses, nurses in training, and nurses not working at the time of the research were not included. According to Kendall’s sample size estimation method (Kendall, 1975), the sample content of descriptive research is 5 to 10 times the number of variables, the general demographic information questionnaire of this study has a total of 13 socio-demographic variables and four scales with 18 dimensions, a total of 31 variables, considering a 20% questionnaire failure rate, the estimated sample size is 186 to 372 cases. In the end, 398 questionnaires were sent out, 356 responses were received, and the response rate was 89.45%. Nurses who participated in the surveys covered surgery, gynecology, intensive care, emergency, operating room, and other clinical departments.

### 2.4. Questionnaire tool

#### 2.4.1. Demographic variables

A self-made demographic questionnaire was utilized in this study to collect the characteristics of participants, including gender, age, educational background, and length of nursing work.

#### 2.4.2. Psychological resilience

Connor-Davidson Resilience Scale (CD-RISC) was published by American scholars Connor and Davidson (2003) in 2003 and has been translated into multiple languages. The Conner-Davidson Scale (CD-RISC) was translated by Chinese scholars (Li et al., 2014) and tested for reliability and validity in a group of nurses, and the results showed that the scale has good psychometrics and can be used to measure nurses’ psychological resilience. There are 25 items on the scale (e.g., I can adapt to change) with 3 dimensions: resilience (13 items), self-improvement (8 items), and optimism (4 items). The Likert 5-point scale was used, with 0–4 being “incorrect,” “rarely correct,” “sometimes correct,” “usually correct,” and “correct.” “The higher the score, the better the resilience of the nurse to face difficulties and challenges. The total Cronbach’s α-value of this scale was 0.930 in the previous study. The CD-RISC overall Cronbach’s α-value was 0.966 in this study.

#### 2.4.3. Career identity

The Nurses’ Career Identity Scale (NCIS) was developed by Ling Liu (Liu et al., 2011), which has 30 items (e.g., Nursing gives me a sense of worth) with 5 dimensions: professional cognitive evaluation (nine items), professional social skills (six items), professional social support (six items), professional frustration coping (six items), and professional self-reflection (three items). The Likert 5-point scale was used, with “1–5″ representing “very non-conforming “to “very conforming “The higher the score, the higher the level of career identity. The total Cronbach’s α-value of this scale was 0.938 in the previous study. The total Cronbach’s α-value was 0.971 in this study. The scale has good reliability and validity.

#### 2.4.4. Work-related quality of life

The Work-Related Quality of Life Scale (WRQOL-2) (Van Laar et al., 2007), version 2, was used in this study. The scale has good reliability after cultural debugging by Shao et al. (2014). The scale consisted of 33 items (e.g., The leadership of the organization provides me with the necessary conditions to work effectively) and 7 dimensions, including work conditions (six items), work stress (five items), work control (five items), work-family balance (two items), work evaluation (five items), general well-being (five items), and career satisfaction (five items). The Likert 5-point scale was used, with “1–5” representing “strongly disagree” to “strongly agree.” The score is 33–165 points. The work stress dimension is an inverse item, and the score of the corresponding item should be subtracted from 6. The total Cronbach’s α-value of this scale was 0.939 in a previous study. The WRQOL-2 overall Cronbach’s α-value was 0.958 in this study.

#### 2.4.5. Work engagement

The 15-item Utrecht Work Engagement Scale developed by Schaufeli et al. (2016), a foreign scholar, is the most widely used scale for measuring occupational work engagement in different cultures and regions. In this research, Zhang and Gan (2005) translated and modified the Chinese version of the Work Engagement Scale was used as a research tool. The scale consisted of 15 items (e.g., I’m passionate about my work) and three dimensions, including energy (six items), dedication (four items), and absorption (five items). The Likert 7-point scale is used to rate each item, with 0 representing “never” and 6 representing “always.” The higher the score, the more engaged the nurses are at work. The Cronbach’s α-value of this scale was 0.735–0.767 in a previous study. The total Cronbach’s α-value of this scale in this study was 0.961.

### 2.5. Data collection

The surveys were powered by www.wjx.cn (a reputable online platform for surveys, ratings, and voting) and were made available via a link on WeChat, a free application for instant messaging services. The WeChat link was shared with the participants with the help of the nurse managers at the hospital, and they were instructed to complete the survey within 2 weeks. The survey guide included information about the questionnaire’s objective, methodology, substance, and inclusion criteria. It was considered that participants who clicked on the link and finished the survey gave their consent to participate in the study. We have taken various precautions to prevent the downsides of online surveys to ensure attentive and accurate completion. For instance, answers that took less than 5 min to complete and were highly inconsistent with personal information were not included. The questionnaire was also completed anonymously with a restriction (each IP address could only be used once).

### 2.6. Data analysis

The association between psychological resilience and work engagement as well as the mediating role of career identity and quality of work life on these two variables were both confirmed by the data analysis that followed. Firstly, the statistical description of the count data is expressed as a component ratio [*n* (%)], and the measured data by the normal distribution is expressed as (*M*±SD). Using an independent sample t-test or one-way ANOVA, this paper analyses the influence of demographic characteristics on work engagement. Secondly, Pearson Correlation Analysis was used to compare the correlations of psychological resilience, professional identity, work quality, and work engagement. Thirdly, Process V3.5 in SPSS (IBM. V25.0) was used to investigate the mediation effects of career identity and work-life quality on psychological resilience and job engagement. Finally, Using the bootstrap method, the 95% confidence interval was estimated by 5,000 times, and the Two-sided inspection level α =0.05. The confidence interval did not include a zero, suggesting statistical significance.

### 2.7. Ethical considerations

The ethical committees of all the participating hospitals gave this study their blessing (approval number JZMULL2023053). Before collecting data, confidentiality norms and protocols established in the Declaration of Helsinki were respected. All participants gave their informed consent.

## 3. Results

### 3.1. General characteristics of the study

The results of this study showed that almost all the nurses were female. Most of the nurses were under 40 years old. Most of them had a bachelor’s degree. More than three-quarters of nurses are married. Most of the nurses had worked for more than 6 years. Most of the nurses were employed on a contract basis. Most of them had the junior or above title of nurse practitioner. Nearly half of the nurses had to work 6 to 9-night shifts per month. For about half of the nurses, the average monthly gross income was between 4,000 and 5,999 yuan. Few nurses considered nursing work as their pursuit. However, there was no statistical difference in the work engagement of nurses of different sexes, ages, educational backgrounds, marriage status, only child and number of offspring, (*p* > 0.05). See Table 1 for details.

**Table 1:** Baseline characteristics and differences in the work engagement score of nurses (*N* = 356).

Variables	Frequency	Percentage	Work engagement scale (*M*±SD)	*t/F*	*p*
*Gender*				−1.550	0.122
Female	343	96.3%	47.72±18.20		
Male	13	3.7%	55.69±18.43		
*Age (years)*				3.322	0.006
<25	14	3.9%	44.14±18.68		
25 ~ 30	99	27.8%	45.24±16.725		
31 ~ 35	103	28.9%	45.46±18.52		
36 ~ 40	76	21.3%	53.87±18.867		
41 ~ 45	35	9.8%	47.11±19.35		
≥46	29	8.1%	54.10±15.18		
*Educational background*				0.355	0.702
Junior college or below	49	13.8%	46.63±17.741		
Bachelor’s	277	77.8%	48.01±18.539		
Master’s or above	30	8.4%	50.2±16.552		
*Working experience (years)*				4.711	<0.001
1 ~ 5	83	23.3%	45.28±16.53		
6 ~ 10	87	24.4%	46.09±18.10		
11 ~ 15	124	34.8%	47.34±18.97		
16 ~ 20	25	7.0%	62.32±14.98		
21 ~ 25	15	4.2%	45.07±18.62		
≥26	22	6.2%	55.41±12.03		
*Marital status*				1.137	0.322
Unmarried	92	25.8%	45.58±16.25		
Married	253	71.1%	48.92±18.94		
Other^a^	11	3.1%	47.45±17.28		
*Only child*				0.742	0.291
Yes	193	54.2%	48.67±18.85		
No	163	45.8%	47.23±17.53		
*Number of offspring*				2.872	0.058
Zero	135	37.9%	45.07±16.65		
One	185	52.0%	49.67±18.84		
Two or more	36	10.1%	50.47±19.84		
*Work department*				10.096	<0.001
internal medicine	146	41.0%	44.07±17.43		
Surgery	65	18.3%	42.15±17.08		
ICU or emergency	70	19.7%	52.76±18.27		
Operating room	40	11.2%	59.63±18. 93		
Other	35	9.8%	52.54±13.28		
*Employment Method*				3.574	0.029
Contract system	268	75.3%	46.86±18.57		
Personnel agent	4	1.1%	66.25±15.88		
Officially on staff	84	23.6%	50.81±16.64		
*Professional title*				3.375	0.035
No title	67	18.8%	46.93±18.90		
Junior tile	153	43.0%	45.71±17.72		
Intermediate title	114	32.0%	51.53±17.71		
Deputy senior	22	6.2%	49.09±20.76		
*Monthly Number of night shifts(days)*				6.324	<0.001
0	96	27.0%	51.69±16.16		
1 ~ 5	89	25.0%	52.08±18.39		
6 ~ 9	140	39.3%	44.42±18.94		
≥10	31	8.7%	41.13±15.94		
*Monthly income*				5.252	0.001
<4,000	106	29.8%	43.32±17.983		
4,000 ~ 5,999	183	51.4%	50.09±17.84		
6,000 ~ 7,999	53	14.9%	47.11±18.619		
≥8,000	14	3.9%	59.64±15.69		
*Reasons for choosing nursing work*				9.872	<0.001
Personal passion	71	19.9%	58.65±18.25		
Listening to others	85	23.9%	46.96±17.80		
Accepting transfers	11	3.1%	41.09±14.13		
Easily employable	110	30.9%	47.23±17.25		
Other	79	22.2%	41.62±16.74		

a Including divorced and widowed participants.

### 3.2. Correlation analysis of major study variable

The Pearson correlation analysis indicated that psychological resilience was positively correlated with career identity ( r =0.723, *p* <0.01), quality of life ( r =0.731, *p* <0.01), and work engagement ( r =0.702, *p* <0.01). In the same way, there was a positive correlation between career identity and quality of work life ( r =0.803, *p* < 0.01) and work engagement ( r =0. 766, *p* <0.01). Moreover, the quality of work life was positively associated with work engagement. ( r =0.749, *p* <0.01). See Table 2 for details.

**Table 2:** Correlation analysis.

Variables	Mean	SD	1	2	3	4
1. psychological resilience	59.95	17.691	–			
2. Career identity	101.34	21.781	0.723^**^	–		
3. Quality of work life	109.24	25.004	0.731^**^	0.803^**^	–	
4. work engagement	48.01	18.243	0.702^**^	0.766^**^	0.749^**^	–

SD =standard deviation. ^**^p <0.01.

### 3.3. Multiple mediating analyses between variables of clinical nurse

After controlling the factors such as sex, age, educational background, work experience, and other eleven factors, the results revealed that the total effect of psychological resilience on work engagement ( β =0.6774, t =17.6889, *p* <0.001) and the direct effect ( β =0.2214, t =4.4338, *p* <0.001) was both significant. Psychological resilience is a significant predictor of career identity ( β = 0.8563, t =19.3529, *p* <0.001). Career identity predicts work engagement ( β =0.2782, t =5.8605, *p* < 0.001), which suggests that career identity plays a mediating role in psychological resilience and work engagement. Similarly, psychological resilience significantly predicts quality of work life ( β =0.4431, t =7.0267, *p* <0.001) and quality of work life predicts work engagement ( β =0.2163, t =5.3873, *p* <0.001), indicating that quality of work life played a mediating role between psychological resilience and work engagement. In addition, career identity can also significantly predict the quality of work life ( β =0.6585, t =12.3608, *p* <0.001). Thus, career identity and the quality of work life had a chain mediation effect on the psychological resilience of the nurses in China. See Table 3 for details.

**Table 3:** Regression model of the effect of psychological resilience on work engagement among nurses in China.

Variables	β	t	P	LLCI	ULCI	R^2^	*F*
Step 1 Outcome variable: career identity							
Predictor psychological resilience	0.8563	19.3529	0.000	0.7693	0.9434	0.5922	35.3781
Step 2 Outcome variable: quality of work life							
Predictor Psychological resilience	0.4431	7.0267	0.000	0.3190	0.5671	0.7015	53.2565
Mediator Career identity	0.6585	12.3608	0.000	0.5537	0.7633		
Step 3 Outcome variable: work engagement							
Predictor Psychological resilience	0.2214	4.4338	0.000	0.1232	0.3197	0.6936	47.9727
Mediator 1 Career identity	0.2782	5.8605	0.000	0.1848	0.3716		
Mediator 2 Quality of work life	0.2163	5.3873	0.000	0.1373	0.2952		
Step 4 Outcome variable: work engagement							
Independent variable Psychological resilience	0.6774	17.6889	0.000	0.6021	0.7528	0.5646	31.5839

**Figure 1:**
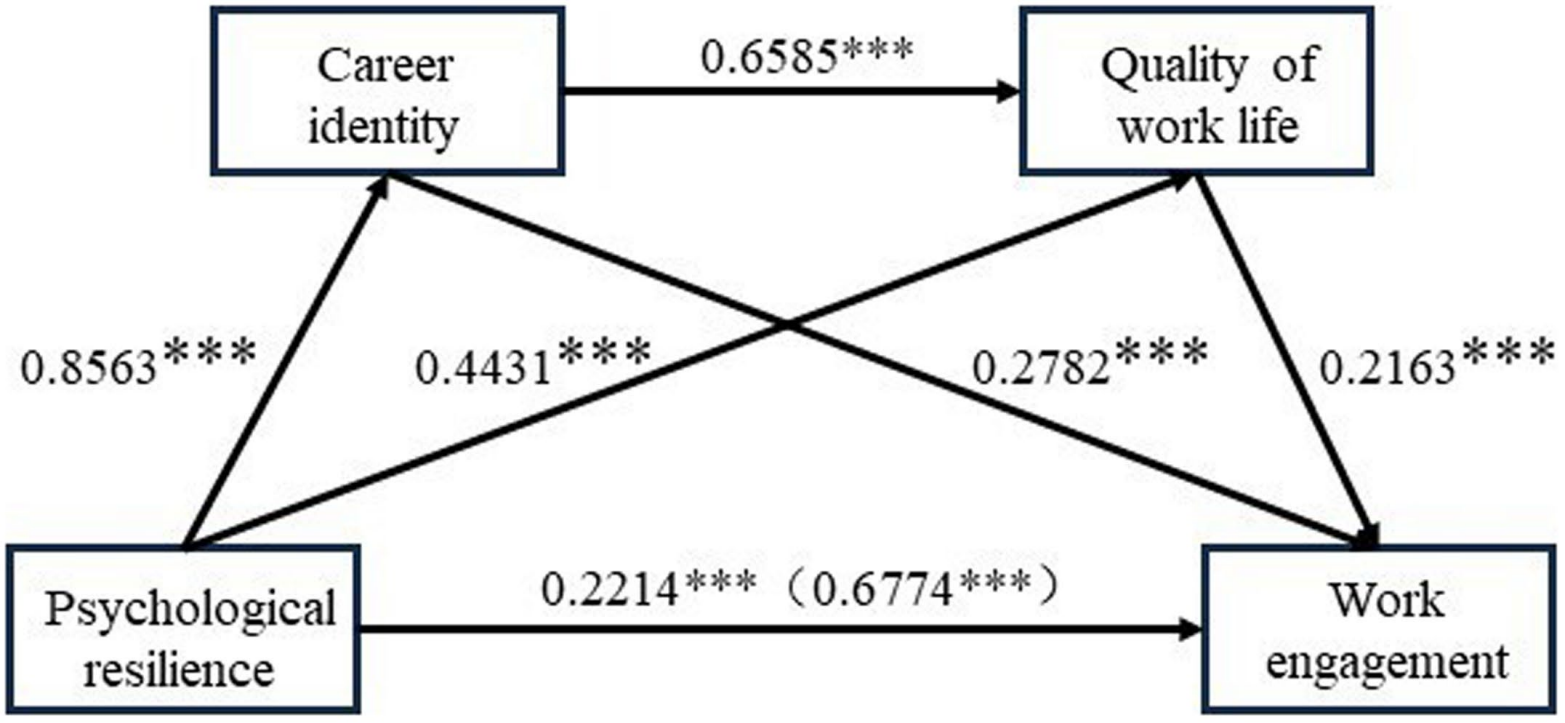
Chain mediating model (^***^p < 0.01).

Table 4‘s mediating effect analysis findings revealed that the total indirect impact’s 95% confidence interval (CI) did not contain zero [Bootstrap 95% CI: 0.3681, 0.5486], representing 67.32% of the total effect. It’s significant to note that three indirect effect pathways had an impact on the connection between psychological resilience and work engagement. First, Path 1’s mediating effect value (psychological resilience →career identity → work engagement) was 0.2382 [Bootstrap 95% CI : 0.1543, 0.3392], representing 35.16% of the total effect. Secondly, Path2’s mediating effect value (psychological resilience → quality of work life → work engagement) was 0.0958 [Bootstrap 95% CI :0.0424, 0.1537], representing 14.14% of the total effect. Thirdly, Path3’s mediating effect value (psychological resilience → career identity → quality of work life → work engagement) was 0.1219 [Bootstrap 95% CI:0.0684, 0.1880], representing 18% of the total effect. Take note that Figure 1 depicts the chain mediating model.

**Table 4:** Multiple mediated analysis between variables of nurses in China.

Effect types	Effect	Boot SE	Boot LLCI	Boot ULCI	Effect ratio
Total effect	0.6774	0.0383	0.6021	0.7528	100%
Direct effect	0.2214	0.0499	0.1232	0.3197	32.68%
Total indirect effect	0.4560	0.0460	0.3681	0.5486	67.32%
Path 1 Psychological resilience → Career identity → Work engagement	0.2382	0.0476	0.1543	0.3392	35.16%
Path 2 Psychological resilience → Quality of work life → Work engagement	0.0958	0.0283	0.0424	0.1537	14.14%
Path 3 Psychological resilience → Career identity → Quality of work life →Work engagement	0.1219	0.0307	0.0684	0.1880	18%
Comparsion1 (Ind1 minus Ind2)	0.1424	0.0682	0.0182	0.2856	
Comparsion2 (Ind1 minus Ind3)	0.1163	0.0626	−0.0035	0.2454	
Comparsion3 (Ind2 minus Ind3)	−0.0261	0.0366	−0.1073	0.0365	

To determine whether these paths were significantly different from one another, a pairwise analysis of the several indirect effects paths was used. To conclude, Comparison 1 [95% CI: - 0.0182, 0.2856] was statistically significant, and Comparison 2 [Bootstrap 95% CI: −0.0035, 0.2454] and 3 [Bootstrap 95% CI: −0.1073, 0.0365] were not statistically significant. See Table 4 for details.

### 3.4. Model fitness indices

Table 5 shows the values of the model fit indicators utilized in this investigation. The model fitting results show that CFI = 0.953; GFI = 0.872; AGFI = 0.830; RMSEA = 0.085, the judgment value is well-fitted, and the model is acceptable.

**Table 5:** The model fit index (*N* = 356).

Index	*p*	CFI	GFI	AGFI	RMSEA
Reference standard	<0.05	>0.90	>0.90	>0.90	<0.080
Actual value	<0.001	0.953	0.872	0.830	0.085

## 4. Discussion

Based on a critical review of the literature, no researcher has studied the associations and specific pathways of Chinese nurses regarding the four components of psychological resilience, career identity, quality of work life, and work engagement. The current study used a chain mediating model to examine how psychological resilience, career identity, and quality of work life affect Chinese nurses’ work engagement. The results confirmed the hypothesis and verified the mediating roles of career identity and quality of work life in the connection between psychological resilience and work engagement.

This study’s primary finding that caught my attention was that, after controlling for gender, age, educational background, work experience, marital status, only child, number of offspring, work department, employment method, professional title, the monthly number of night shifts, monthly income, and reasons for choosing nursing work, psychological resilience still had a significant positive impact on work engagement. The findings of this study are consistent with those of the study on the relationship between work engagement and the psychological resilience of hemodialysis nurses (Cao and Chen, 2019). We expanded this finding to clinical nurses in China. Due to China’s expanding health services, the number of nurses is growing. As a result of the massive influx of ill patients, workplace violence (Lu et al., 2019), and inadequate funding for healthcare services, such as a shortage of beds and an imbalance between patients and healthcare professionals (Zeng et al., 2013), nurses were under increasing physical and psychological strain. These factors contribute to the occurrence of burnout in the nursing population and reduce nurses’ work engagement in their work. However, nurses who have high levels of psychological resilience can cope well with stress at work, have high levels of job satisfaction, and have better overall health (Zheng et al., 2017; Brown et al., 2018). These factors enable nurses to be better engaged in their work. According to the Job Demands-Resources model (Bakker and Demerouti, 2007), we can better understand that resilience can be a work resource in nursing practice that can enable nurses to unlock their full potential and achieve positive outcomes in the face of workplace challenges. It improves nurse retention and creates a strong nursing workforce (Moloney et al., 2018). Some studies have shown that social support as a useful work resource can enable better engagement, prevent the emergence of depressive tendencies, promote a good outlook on life, and boost resilience (Hsieh et al., 2016, 2017). It can be combined with psychological resilience and be used to increase nurses’ work engagement. As a result, psychological resilience plays a positive role in promoting nurses’ mental health, work engagement, and turnover intention.

Furthermore, this study has demonstrated a good correlation between career identity, quality of work life, and work engagement. This is consistent with the findings of other scholars (Remegio et al., 2021; Caricati et al., 2022; Xing, 2022). According to the social identity theory, an individual’s identity can influence their thinking, emotions, and conduct (Cao et al., 2016). A high career identity allows nurses to foster positive feelings about their work, increase efficacy in their work, feel a stronger sense of accomplishment and satisfaction, and inspire others to work more enthusiastically. According to research on family physicians, the relationship between quality of work life and presenteeism is mediated by career identity (Lu et al., 2018). The work of Sun and Fu showed that there was a positive correlation between the quality of work life of nurses who had standardized training and job participation and that burnout and career identity played a mediating role (Sun et al., 2022). According to Efraty and Sirgy (1990), employees’ career identities are strongly impacted by their perceptions of the quality of their work life. In this study, nurses’ career identity was significantly and positively correlated with quality of work life. When nurses recognize the value of their profession and have a high level of career identity, they are more likely to gain a sense of accomplishment and have a higher quality of work life. Career identity has been found to act as a mediator between the impacts of numerous factors on work engagement in several kinds of research on nurses, including the influence of career benefit on work engagement (Zhou et al., 2019) and the impact of nurse-physician collaboration on work engagement (Lyu X. et al., 2020). Additionally, the nursing staff will benefit from professional training and enhanced communication skills to reduce burnout and increase engagement (Ercolani et al., 2020).

In this study, the chain mediation influence of career identity and quality of work life in the link between Chinese nurses’ psychological resilience and work engagement is another significant discovery. This is the study’s primary theoretical contribution. The results of the study confirmed the four hypotheses proposed by the researchers. The results showed psychological resilience indirectly affects work engagement through four pathways: career identity, quality of work life, and the chain mediating effect of career identity and quality of work life. Previous research has shown that career identity partly mediates the relationship between resilience and quality of work life. The current study demonstrated career identity played a partial mediating role in the correlation between psychological resilience and work engagement (Path 1), accounting for 35.16% of the total indirect effect, which means that psychological resilience and career identity are important predictors in work engagement. This is congruent with Hirschi’s research findings (Hirschi, 2012). According to a study, when nurses approve their work, the more responsible they are and the more willing they are to commit to it (Sun et al., 2022). This study also demonstrated that work engagement and resilience development are both mediated by the quality of work life (Path 2), which represents 14.14% of the total indirect impact. In the study, the correlation coefficient between the quality of work life and work engagement was 0.702, which reached a highly significant level. The quality of work life is an important personal resource, and it is very important to promote the development of the organization and the achievement of the career. There existed another path (psychological resilience→ career identity→ quality of work life→ work engagement) (Path 3), representing 18% of the total indirect effect, which demonstrated psychological resilience influenced work engagement through career identity and quality of work life. Career identity and quality of work life jointly promote work engagement. The study also demonstrated the direct and indirect effects of psychological resilience on work engagement (Path 4), accounting for 32.68 and 67.32% of the total effect, respectively. The direct and indirect effects of clinical nurses’ psychological resilience on work engagement were significant in this study. Thus, career identity and quality of work life played a partial mediating effect in the study. Finally, increased work engagement can assist nurses in reducing negative feelings, enhancing their quality of work life, and maintaining their professional well-being when they are under intense work and psychological stress. In the future, we can keep researching the elements influencing work engagement at various levels, including those of individuals, organizations, and society.

In a nutshell, the study has enriched the content of positive psychology and has deepened our understanding of the mechanism of psychological resilience in work engagement from the angle of nurses in China. The results show that career identity and quality of work life play a key role in this relationship, which can be used to build and develop nurses’ work in the future.

## 5. Conclusion

Work engagement is an essential characteristic of the challenging profession of nursing. This paper offers some insights into the relationship between psychological resilience and work engagement in China’s clinical nurses. The findings support the hypothesis that psychological resilience is positively related to career identity, quality of work life, and work engagement. This research provides evidence for the relationship between psychological resilience and work engagement in China and the mediation effect of career identity and quality of work life. Additionally, through career identity and quality of work life, psychological resilience may have a direct or indirect impact on work engagement. Path 1 was crucial to the overall indirect effect, according to the total indirect effects. This study concludes that Career identity and quality of work life played a chain-mediating role between psychological resilience and work engagement in Chinese clinical nurses and that measures should be taken to improve the work engagement of clinical nurses. Nursing managers pay attention to the comprehensive quality of nurses themselves, guide clinical nurses to give full play to their potential and create a positive working atmosphere. Meanwhile, to improve nurses’ work engagement, hospitals should give emotional support to nurse groups, strive to eliminate the phenomenon of attaching importance to medicine but despising nursing, improve the salary guarantee system, provide abundant work resources, and reduce the consumption of nurses’ personal resources.

## 6. Limitations

This study has the following limitations. First of all, the cross-sectional nature of this study made it impossible to determine the exact cause of the link between the variables. Secondly, convenience sampling restricted the survey’s generalizability, which only applied to nurses working in Jinzhou city’s tertiary hospitals. Thirdly, the reliability of the results may be impacted by the use of self-report tools since participants may provide false information or score based on a partial recall of the survey results. Future studies could broaden their focus to include other regions and a big sample size to produce more solid findings.

## Data availability statement

The raw data supporting the conclusions of this article will be made available by the authors, without undue reservation.

## Ethics statement

The studies involving humans were approved by the Jinzhou Medical University. The studies were conducted in accordance with the local legislation and institutional requirements. The participants provided their written informed consent to participate in this study.

## Author contributions

ZM: Writing – original draft, Writing – review & editing. LZ: Writing – review & editing. HZ: Investigation, Writing – review & editing. JW: Investigation, Writing – review & editing.
